# Sequential Dermoscopic, Histopathologic, and Immunopathologic Documentation of Regressing Seborrheic Keratosis: A Multimodal, Time‐Resolved Visual Case Study

**DOI:** 10.1002/ccr3.72274

**Published:** 2026-03-09

**Authors:** Tomoaki Takada

**Affiliations:** ^1^ Sumikawa Takada Dermatology Clinic Sapporo Japan

**Keywords:** amyloid deposition, apoptosis, cytotoxic T cells, dermoscopy, seborrheic keratosis, spontaneous regression

## Abstract

Seborrheic keratosis (SK) typically shows stable behavior, and spontaneous regression is rarely documented. We report a unique case of SK that underwent complete involution within 8 weeks. Sequential clinical and dermoscopic imaging demonstrated a transition from classical SK structures to red‐blue homogeneous areas and, ultimately, to whitish regression zones. Histopathology revealed interface inflammation, keratinocyte degeneration, and epidermal remodeling. Immunohistochemistry confirmed a stepwise regression mechanism involving CD8^+^/TIA‐1^+^ cytotoxic T‐cell activation, cleaved caspase‐3–mediated apoptosis, and keratinocyte‐derived amyloid deposition. To our knowledge, this is the first case to demonstrate a fully time‐resolved clinicodermoscopic‐immunopathologic sequence of cytotoxic regression in SK.

## Key Clinical Message

Seborrheic keratosis may undergo rapid spontaneous regression driven by cytotoxic T‐cell activation. Sequential dermoscopy and immunopathology revealed a stepwise process involving keratinocyte apoptosis and keratinocyte‐derived amyloid deposition.

## 1 Introduction

Seborrheic keratosis (SK) is one of the most common benign epidermal tumors and typically demonstrates a stable clinical course. Although inflamed or irritated variants are occasionally encountered, spontaneous regression is considered uncommon. Recent dermoscopic studies have suggested that a subset of SKs may undergo partial or complete regression and, in some cases, show features overlapping with lichenoid keratosis (LK), indicating a dynamic spectrum rather than a static entity [1, 2].

Furthermore, the biological processes contributing to cutaneous regression—including cytotoxic T‐cell activation, apoptosis, and subsequent keratinocyte‐derived amyloid deposition—have been described mainly in cutaneous amyloidosis [3], although their relevance to SK remains unclear.

Here, we present a unique case of SK that demonstrated a well‐documented involution sequence supported by longitudinal dermoscopy, histopathology, and immunohistochemistry. To our knowledge, no previous study has demonstrated such a complete temporal correlation in a single SK lesion.

## 2 Case History/Examination

Although biopsy‐induced regression cannot be completely excluded, dermoscopic signs of inflammation were already present before biopsy, suggesting that the regression process had likely begun prior to the procedure.

The lesion had been present for approximately 2 years prior to presentation, with gradual enlargement during this period.

**FIGURE 1:**
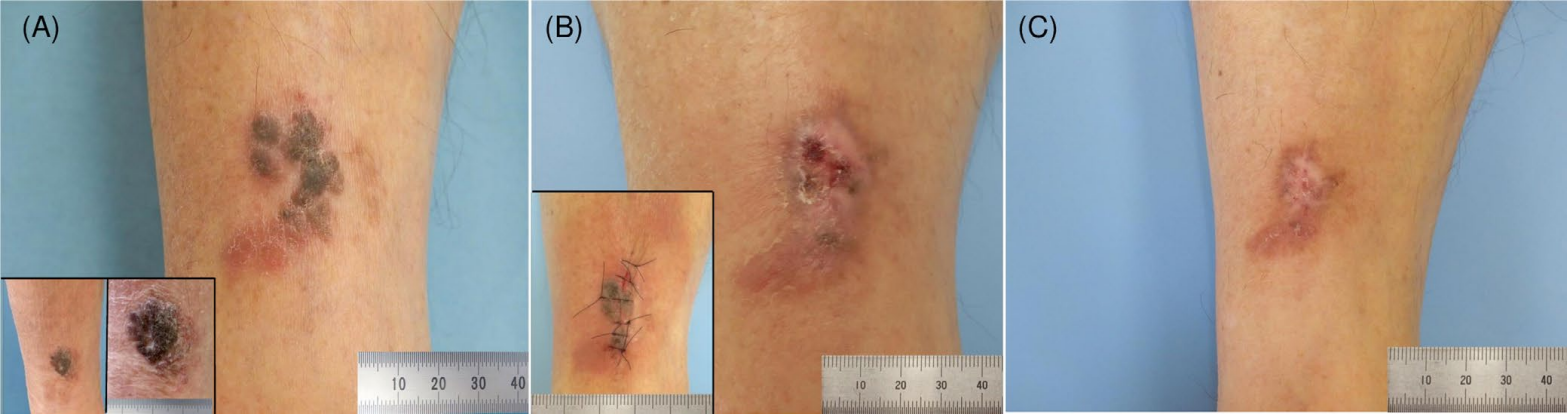
Clinical appearance at baseline, 4 weeks, and 8 weeks after biopsy. This figure demonstrates the sequential clinical changes associated with the spontaneous regression of seborrheic keratosis. (A) Before biopsy, the lesion presented as a lobulated, dark‐brown keratotic plaque with surrounding erythema, partially flattened areas, and a multisegmented configuration. The black boxed inset shows clinical images from 2 years earlier, when the lesion measured 15 × 18 mm. At the time of the biopsy, it had enlarged to 20 × 26 mm. (B) At 4 weeks post‐biopsy, crusting and a red‐bluish to red‐black discoloration reflected active inflammation, hemorrhage, and early regression. The black boxed inset shows images immediately post‐biopsy. (C) At 8 weeks, the lesion was markedly flattened, showing whitish and faintly erythematous areas with minimal residual pigmentation, which is consistent with late‐stage regression and remodeling.

An 86‐year‐old man presented with a brown, slightly elevated lesion on the right lower leg (Figure 1A). At 4 weeks after biopsy, the lesion showed crusting and red‐bluish discoloration corresponding to active regression (Figure 1B). At 8 weeks, marked flattening with whitish regression areas was observed (Figure 1C).

**FIGURE 2:**
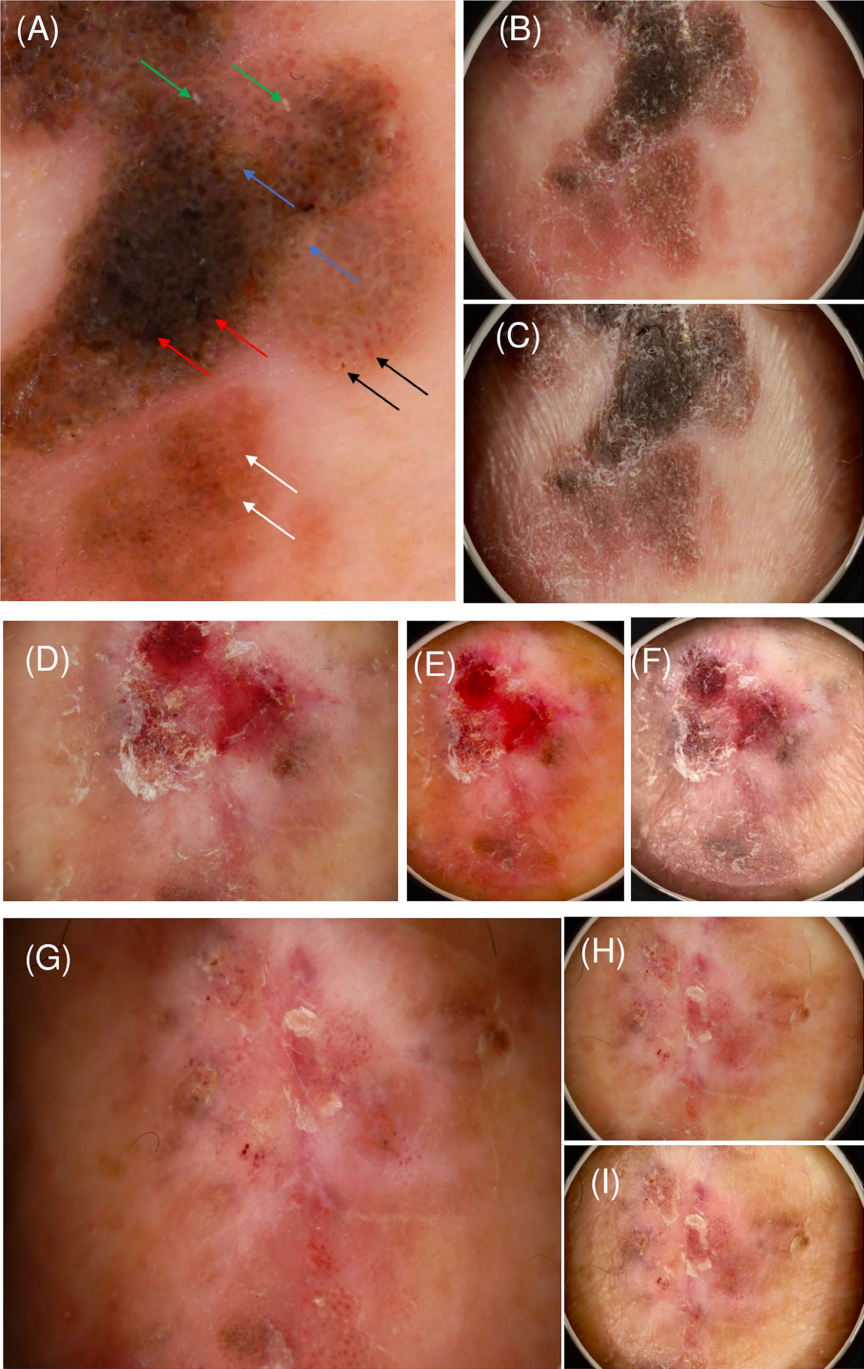
Dermoscopic findings at baseline, 4 weeks, and 8 weeks using three imaging modes. This figure illustrates the chronological changes in the dermoscopic features of the lesion, tracked using three distinct imaging modes throughout the regression process. Description of imaging modes: DERMO mode (Panels A, D, G): This mode primarily highlights superficial keratin structures, including milia‐like cysts, comedo‐like openings, and fissure–ridge patterns. DERMO CONT polarized mode (Panels B, E, H): This mode visualizes larger inflammation‐related changes, such as red–blue homogeneous areas and milky‐red zones. DERMO CONT non‐polarized mode (Panels C, F, I): This mode is used to show fine whitish/gray regression structures and residual pigmentation. Chronological findings: (A–C) Baseline: The images show classical seborrheic keratosis features along with early signs of regression. Small pinkish round structures (white arrows), hairpin vessels with a white halo (black arrows), and fissures and ridges (red arrows) are observed. Milia‐like cysts (green arrows) and comedo‐like openings (blue arrows) are clearly distinguished. (D–F) At 4 weeks: Active regression is progressing, characterized by the expansion of irregular red/blue homogeneous areas. The fissure and ridge structures are actively collapsing. (G–I) At 8 weeks: The lesion is largely replaced by whitish/grayish regression structures, with only minimal residual pigmentation remaining.

Dermoscopy revealed classical SK structures, including milia‐like cysts and comedo‐like openings (Figure 2A–C). After 4 weeks, the lesion exhibited red‐blue homogeneous areas with brown‐gray granules, corresponding to an intermediate regression stage (Figure 2D–F). At 8 weeks, whitish structureless regression areas predominated (Figure 2G–I).

**FIGURE 3:**
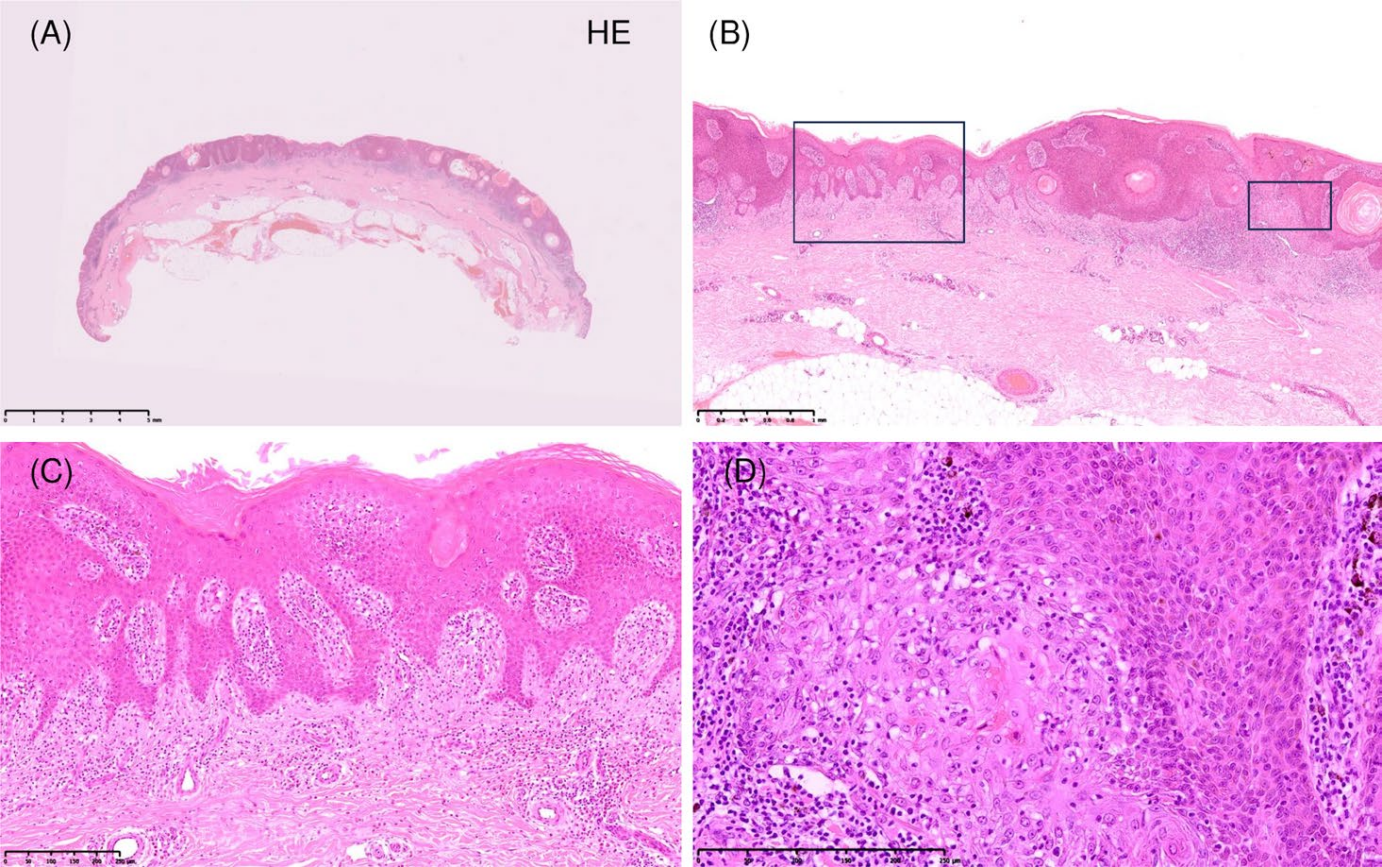
Histopathologic findings (H&E staining). This figure presents the key histological features of the lesion at the time of biopsy, confirming findings suggestive of regression. (A) The low‐power overview shows the horizontally elongated morphology of the lesion. Original magnification: ×5; scale bar: 5 mm. (B) A transition from flattened to elevated epidermis is observed, accompanied by inflammatory infiltrates. Original magnification: ×25; scale bar: 1 mm. (C) Basaloid and squamoid proliferation is present, along with pseudo–horn cysts and superficial dermal fibrosis. Original magnification: ×100; scale bar: 250 μm. (D) Necrotic epithelial nests with dense lymphocytic infiltration are confirmed, representing the active regression phase. Original magnification: ×200; scale bar: 250 μm.

**FIGURE 4:**
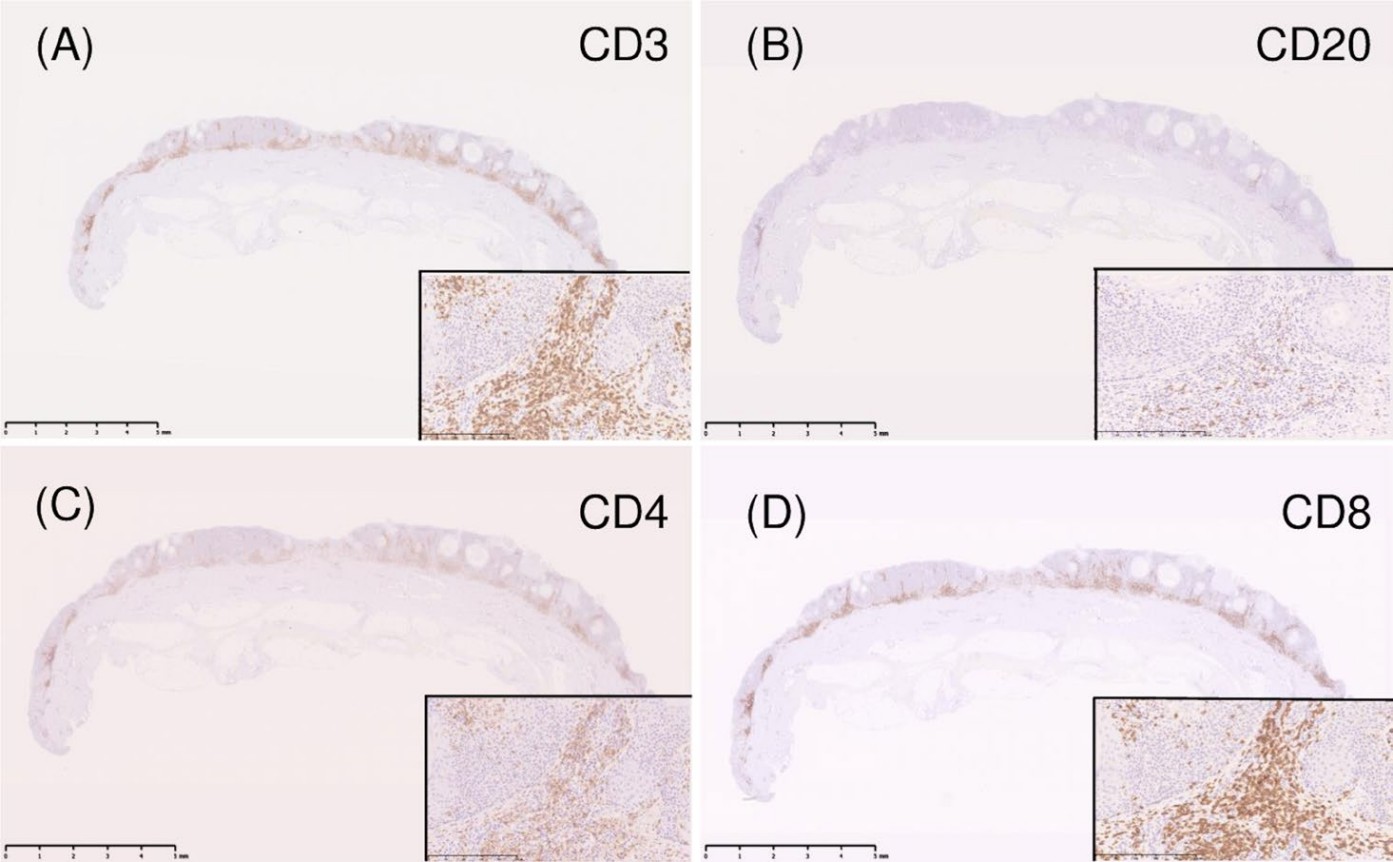
Lymphocytic immunophenotype. This figure evaluates the immunohistological phenotype of the lymphocytic infiltrate within the lesion. (A) Immunostaining for CD3 (a T‐cell marker) highlights a diffuse T‐cell infiltration within the lesion. (B) Immunostaining for CD20 shows only a sparse presence of B‐cells. (C) CD4‐positive T cells are present, but their numbers are fewer compared to CD8‐positive cells. (D) CD8‐positive cytotoxic T cells (the effector cells of regression) are observed to predominate around the degenerating keratinocytes. Magnification: The overall view for (A)–(D) is ×5 (scale bar: 5 mm). Insets show higher magnification views (×200, scale bar: 250 μm).

**FIGURE 5:**
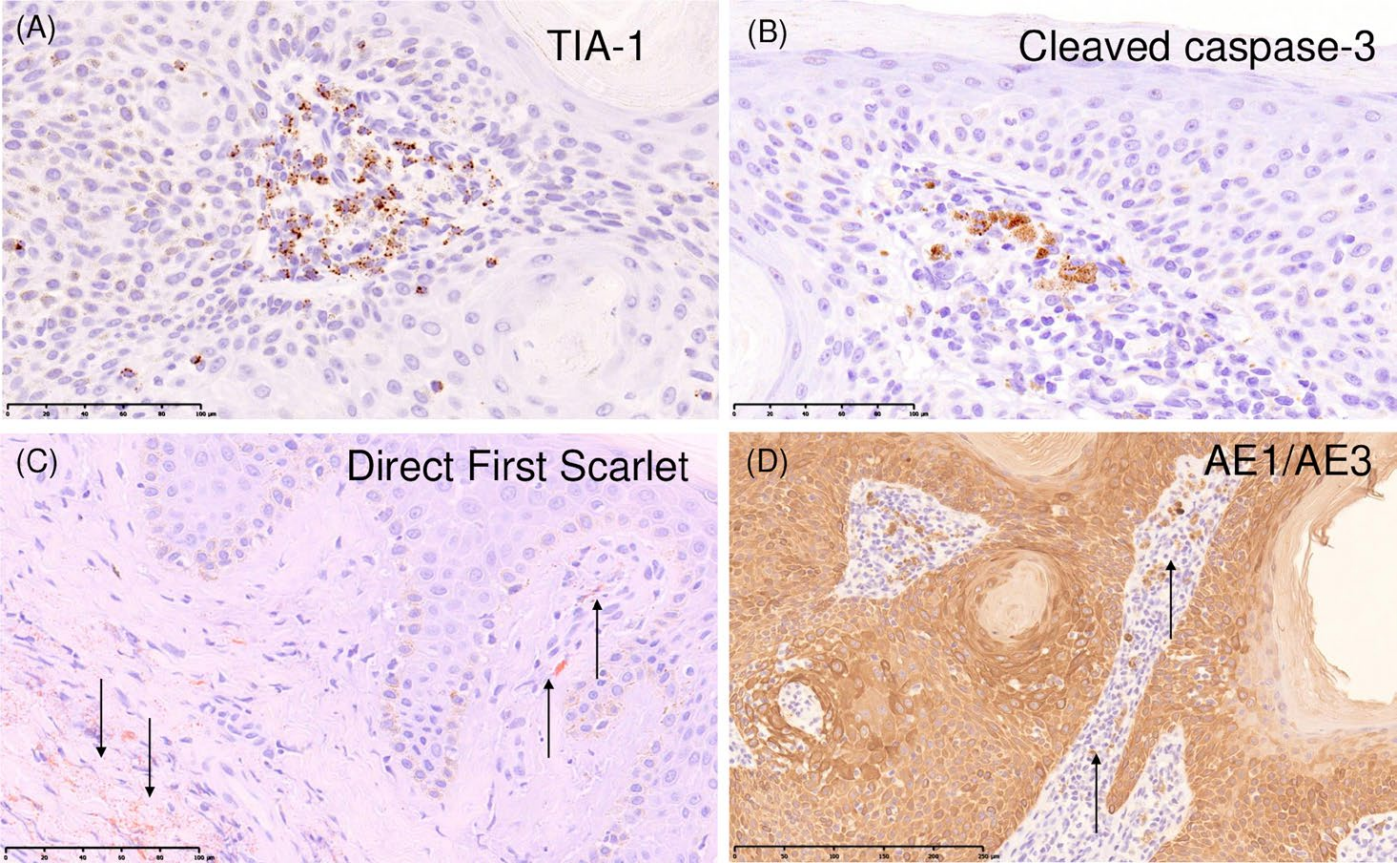
Cytotoxic activation, apoptosis, and keratinocyte‐derived amyloid. This figure provides molecular and cellular evidence related to the mechanism of regression in seborrheic keratosis. (A) TIA‐1 staining demonstrates cytotoxic granule–positive lymphocytes positioned adjacent to the damaged keratinocytes. Magnification: ×400; scale bar: 100 μm. (B) Cleaved Caspase‐3 staining reveals widespread apoptosis within the keratinocytes. Magnification: ×400; scale bar: 100 μm. (C) Direct Fast Scarlet (DFS) staining highlights the presence of finely granular amyloid deposits (black arrows). Magnification: ×400; scale bar: 100 μm. (D) AE1/AE3 staining shows focal positivity in the dermal deposits (black arrows), confirming that this material is keratinocyte‐derived. Magnification: ×200; scale bar: 250 μm.

Histopathology showed interface dermatitis with basal vacuolar changes and band‐like lymphocytic infiltration (Figure 3). Immunohistochemistry demonstrated predominant CD8^+^ (Figure 4) and TIA‐1^+^ cytotoxic T cells (Figure 5A) and numerous cleaved caspase‐3–positive apoptotic keratinocytes (Figure 5B). Direct Fast Scarlet–stained (Figure 5C) eosinophilic deposits and AE1/AE3 immunostaining (Figure 5D) confirmed keratinocyte‐derived amyloid.

A diagnostic partial biopsy was performed at baseline to confirm the nature of the lesion. Although trauma‐induced regression has been described in inflamed SK, the subsequent changes in our case were diffuse and extended beyond the biopsy area. This suggests that the observed involution process primarily reflects spontaneous regression rather than a biopsy‐related change.

Dermoscopy was performed using a DERMO‐CONT device in both polarized and non‐polarized modes at baseline, 4 weeks, and 8 weeks. Standard hematoxylin–eosin staining and immunohistochemical analyses including CD4, CD8, TIA‐1, and cleaved caspase‐3 were performed. Direct Fast Scarlet and AE1/AE3 staining were used to identify keratinocyte‐derived amyloid.

## 3 Differential Diagnosis, Investigations and Treatment

The differential diagnosis included inflamed SK, lichenoid keratosis, and regressing melanoma. A diagnostic partial biopsy was performed to confirm the benign nature of the lesion. Histopathologic and immunohistochemical analyses were conducted as described above. No additional treatment was administered after biopsy.

## 4 Conclusion and Results (Outcome and Follow‐Up)

This case illustrates a time‐resolved regression pathway of SK driven by cytotoxic T‐cell activation, keratinocyte apoptosis, and keratinocyte‐derived amyloid deposition. This case highlights a novel, time‐resolved sequence of SK regression supported by integrated dermoscopic and immunopathologic findings. No recurrence has been detected during follow‐up (Figure 1C). The lesion underwent complete flattening with residual whitish regression areas by Week 8. This case provides an educational visual framework demonstrating how seborrheic keratosis can undergo stepwise regression over time, thereby helping clinicians avoid misdiagnosis of regressing lesions.

## 5 Discussion

SK is typically regarded as a benign epidermal tumor with a stable clinical course, and spontaneous regression is considered uncommon. However, accumulating evidence indicates that a subset of SKs may undergo partial or complete involution. Hiraiwa et al. reported two cases showing partial spontaneous regression of SK [4], and earlier observations by Furue et al. described spontaneous regression of multiple lesions in association with systemic conditions [5]. These reports support the notion that SK can enter a regressive phase, although the mechanisms and chronological sequence remain insufficiently characterized.

Dermoscopy‐based follow‐up studies have further suggested that SK may progress through intermediate inflammatory stages resembling lichenoid keratosis (LK). Zaballos et al. demonstrated that SKs evolving toward LK exhibit brown‐gray granules, red‐blue areas, and whitish regression structures [1, 2]. However, prior dermoscopic studies lacked integration with histopathologic or immunologic correlation within a single lesion.

In the present case, we were able to document—at three clearly separated time points—a continuous sequence from classical SK morphology to LK‐like inflammatory changes and finally to complete regression. Dermoscopy showed the expected transition from milia‐like cysts and comedo‐like openings to red‐blue inflammatory areas and, ultimately, whitish regression zones. Histopathology revealed interface dermatitis, basal vacuolar alteration, and dense lymphocytic infiltration, corresponding to the clinical and dermoscopic changes.

Importantly, immunohistochemical analysis demonstrated a predominance of CD8^+^ and TIA‐1^+^ cytotoxic T cells adjacent to degenerating keratinocytes, together with numerous cleaved caspase‐3–positive apoptotic cells. This cytotoxic immune profile parallels the mechanisms described in regressing lichenoid keratosis, keratoacanthoma, and other benign regressive processes, as reported by Bayer‐Garner et al. [6]. The presence of keratinocyte‐derived amyloid, confirmed by Direct Fast Scarlet staining and AE1/AE3 immunoreactivity, further supports a pathway in which cytotoxic injury induces keratinocyte apoptosis and subsequent amyloid deposition. Although amyloid formation has been well documented in primary cutaneous amyloidosis [3], its demonstration in a regressing SK has been rare. We acknowledge that AE1/AE3 positivity should be interpreted cautiously, as it may stain keratin‐derived material. In the present case, this finding was used to support, but not overinterpret, the presence of keratinocyte‐derived amyloid during regression.

Previous follow‐up studies, including the short dermoscopic series by Fikrle [7], have illustrated structural evolution in regressing SKs, but none have provided a fully time‐resolved and multimodal correlation across dermoscopy, histopathology, and immunophenotyping. The present case therefore represents the first complete clinicodermoscopic‐histopathologic‐immunologic documentation of SK regression within a single lesion.

Taken together, our findings support the concept that LK represents an intermediate inflammatory stage within a dynamic regression spectrum of SK, driven by cytotoxic T‐cell–mediated keratinocyte injury and remodeling.

## 6 Limitations

This is a single‐case study, and further studies are needed to determine the prevalence and generalizability of this regression mechanism.

## Author Contributions

**Tomoaki Takada:** conceptualization, data curation, formal analysis, funding acquisition, investigation, methodology, project administration, resources, software, supervision, validation, visualization, writing – original draft, writing – review and editing.

## Funding

The author has nothing to report.

## Ethics Statement

Ethical approval was not required for this single‐patient case report because all procedures were performed as routine clinical care and involved no experimental interventions.

## Consent

Written informed consent was obtained from the patient for the publication of this case report and the accompanying images.

## Conflicts of Interest

The author declares no conflicts of interest.

## Data Availability

Data sharing is not applicable to this article as no datasets were generated or analyzed beyond routine clinical documentation.
